# Field-Portable Technology for Illicit Drug Discrimination via Deep Learning of Hybridized Reflectance/Fluorescence Spectroscopic Fingerprints

**DOI:** 10.1021/acs.analchem.4c05247

**Published:** 2025-05-07

**Authors:** Alexander Power, Matthew Gardner, Rachael Andrews, Gyles Cozier, Ranjeet Kumar, Tom P. Freeman, Ian S. Blagbrough, Peter Sunderland, Jennifer Scott, Anca Frinculescu, Trevor Shine, Gillian Taylor, Caitlyn Norman, Hervé Ménard, Niamh N. Daéid, Oliver B. Sutcliffe, Stephen M. Husbands, Richard W. Bowman, Tom S. F. Haines, Christopher R. Pudney

**Affiliations:** † Department of Computer Science, 1555 University of Bath, Bath BA2 7AY, U.K.; ‡ Department of Life Sciences, University of Bath, Bath BA2 7AY, U.K.; § Department of Psychology, University of Bath, Bath BA2 7AY, U.K.; ∥ Centre for Academic Primary Care, Bristol Medical School, 1980 University of Bristol, Bristol BS8 2PS, U.K.; ⊥ TICTAC Communications Ltd., 3526 St. George’s University of London, Room 1.159 Jenner Wing, Cranmer Terrace, London SW17 0RE, U.K.; # School of Health and Life Sciences, 5462 Teesside University, Middlesbrough TS1 3BX, U.K.; ¶ Leverhulme Research Centre for Forensic Science, 3042 University of Dundee, Dundee DD1 4HN, U.K.; ∇ MANchester DRug Analysis & Knowledge Exchange (MANDRAKE), Department of Natural Sciences, Manchester Metropolitan University, Manchester M1 5GD, U.K.; ○ School of Physics and Astronomy, University of Glasgow, Glasgow G12 8QQ, U.K.

## Abstract

Novel psychoactive substances (NPS) pose one of the greatest challenges across the illicit drug landscape. They can be highly potent, and coupled with rapid changes in structure, tracking and identifying these drugs is difficult and presents users with a “Russian roulette” if used. Benzodiazepines, synthetic opioids, synthetic cannabinoids, and synthetic cathinones account for the majority of NPS-related deaths and harm. Detecting these drugs with existing field-portable technologies is challenging and has hampered the development of community harm reduction services and interventions. Herein, we demonstrate that hybridizing fluorescence and reflectance spectroscopies can accurately identify NPS and provide concentration information with a focus on benzodiazepines and nitazenes. The discrimination is achieved through a deep learning algorithm trained on a library of preprocessed spectral data. We demonstrate the potential for these measurements to be made using a low-cost, portable device that requires minimal user training. Using this device, we demonstrate the discrimination of 11 benzodiazepines from “street” tablets that include bulking agents and other excipients. We show the detection of complex mixtures of multiple drugs, with the key example of nitazene + benzodiazepine (metonitazene + bromazolam), fentanyl + xylazine, and heroin + nitazene (etonitazene) combinations. These samples represent current drug trends and are associated with drug-related deaths. When combined with the implementation of detection technology in a portable device, these data point to the immediate potential to support harm reduction work in community-based settings. Finally, we demonstrate that the approach may be generalized to other drug classes outside NPS discrimination.

## Graphical abstract

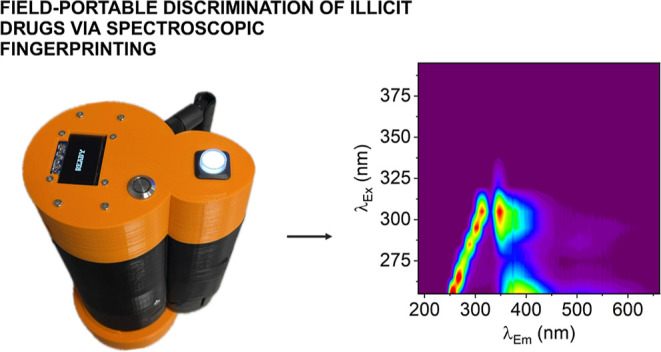

The emergence and differentiation of novel psychoactive substances (NPS)—synthetic drugs of six major classes including stimulants, synthetic cannabinoid receptor agonists, hallucinogens, opioid receptor agonists, sedatives/hypnotics, and dissociatives—is of growing concern.^1^ NPS are structural analogues of classical drugs of abuse. They are known to mimic the physiological effects of these compounds by targeting the same receptors or transporters that mediate their molecular mechanisms.^2,3^ Clandestine synthesis is a major route of NPS production, with the number of clandestine laboratories increasing substantially in recent years.^4^ As NPS are made increasingly available through these routes, their popularity has risen due to low cost, ease of accessibility, and the perception of reduced legal liability.^5^ Given their high potency, unpredictable side effects, and widespread use, the increasing incidence of overdose and drug-related death is perhaps not surprising.^6–8^

As a useful example, benzodiazepines (BZDs) are a prominent class of NPS with increasing trends of abuse and drug-related death, being involved in over two-thirds of drug-related deaths in Scotland in 2022.^9^ So-called “street” BZDs are illicit drugs that fall into two distinct groups, diverted prescription medications licensed for therapeutic use in some countries and designer compounds synthesized by clandestine chemists.^10^ Street BZD use is problematic due to the prevalence of counterfeit medicines, mis-sold in pill form as legitimate pharmaceuticals, where in fact potent NPS benzodiazepines are present in unknown quantities.^11–13^ Etizolam is a notable example of a street BZD that appears in counterfeit medication in the UK, although it is legal in some other countries. Etizolam produces pharmacological effects at ten times the potency of the licensed drug diazepam.^14,15^ People who use these drugs, therefore, are doubly at risk in not knowing the drug or the dose that they are taking.^16^ For example, data from Welsh Emerging Drugs and Identification of Novel Substances (WEDINOS) from June–July 2024, shows 59% of diazepam submissions contained a drug other than diazepam.^17^ Similarly, the UK and other countries have seen the very recent rise of synthetic opioids (nitazenes), with potencies higher than fentanyl^18,19^ and, at the time of writing, are found in combination with BZDs and other counterfeit pharmaceuticals as well as heroin in the UK. This contrasts with the high prevalence of fentanyl in the USA.

Detection of NPS drugs is almost exclusively via lab-based analysis (typically GC–MS). However, to enable community drug checking and testing, ideally one would be able to identify NPS in the field instantly, with minimal technical training and at low cost.^20,21^ A range of technologies have been used for this purpose, including hand-held Raman, near-infrared, and FT-IR. However, these technologies are severely challenged by the typically low concentration of NPS in street samples, the presence of bulking agents and other excipients, fluorescence of the analyte, and complex analyte mixtures.^22,23^

As the example of BZDs shows, there is a need for rapid, nontechnical drug identification and ideally concentration discrimination. We have previously validated that fluorescence spectral fingerprinting [FSF; enumerated excitation–emission matrices (EEMS)] is an effective means to detect synthetic cannabinoids.^24^ We have shown that FSFs can be used to discriminate between individual compounds and structural classes.^24,25^ We hypothesize that the varied heterocyclic core structures of other NPS, including benzodiazepines and nitazenes, render these compounds ideal candidates for detection using this approach.

Here, we demonstrate that by expanding the FSF measurement to include reflectance information, we are able to distinguish individual BZDs and provide information on their concentration. Moreover, we demonstrate that nitazenes can be detected using this approach in the presence of other drugs, including BZDs and seized heroin samples. We demonstrate the implementation of these advances in portable device design.

## Results and Discussion

### Establishing the Potential of FSFs for BZD Discrimination and Enabling Hardware for Their Measurement

Figure 1 shows the FSF for exemplar BZDs from three structural classes represented in the illicit designer BZD market.^26^ From Figure 1, each of the different structural classes produces a different FSF, even within the same class. We note that the similarities within the same structural classes are simple, tending to visually recognizable similarities. However, from these data, it appears that even relatively minor substitutions about the core benzodiazepine group produce a unique FSF. For example, the presence of nitro groups on both clonazepam and flunitrazolam may influence FSF structure due to a strong electron-withdrawing effect, narrowing the energy gap between electronic transitions and shifting fluorescence emission toward the red end of the spectrum.^27^

**Figure 1:**
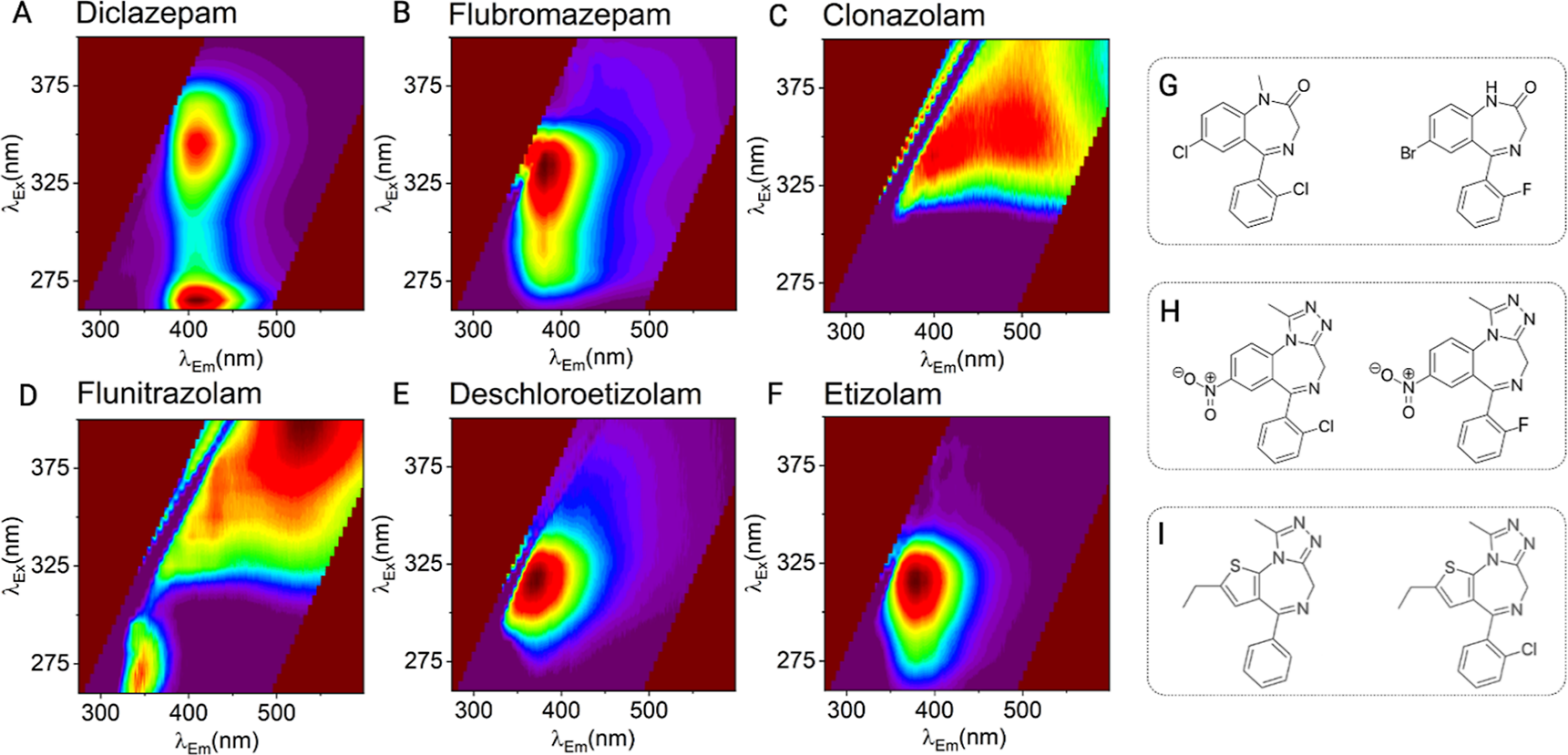
Benzodiazepine structural classes represented in the illicit drug market, with measured fluorescence spectral fingerprints (FSFs) of exemplar compounds. FSFs for 1,4-benzodiazepines (A,B), triazolobenzodiazepines (C,D), and thienotriazolobenzodiazepines (E,F). FSFs measured at 100 μg/mL using a benchtop spectrofluorometer (Edinburgh Instruments, FS5). Excitation (λ_ex_) scanned at 5 nm intervals between 260 and 400 nm and emission (λ_em_) scanned at 0.5 nm intervals between 275 and 600 nm. Recorded in triplicate and background subtracted. Associated chemical structures for 1,4-benzodiazepines (G), triazolobenzodiazepines (H), and thienotriazolodiazepines (I).

These data directly mirror our previous findings with synthetic cannabinoids.^24^ However, we note the fluorophore in BZDs shows a relatively lower quantum yield compared to synthetic cannabinoids (approximately 3 times lower) but differs significantly for individual molecules. Our data therefore suggests that the FSFs for benzodiazepines (Figure 1) could be used to discriminate even structurally similar BZDs.

While FSFs appear to be discriminatory, clearly a benchtop spectrofluorometer (as used to collect data shown in Figure 1) is not a practical solution for community harm reduction, owing to both its size, relative technical complexity to operate, and the lack of inbuilt interpretive software. For this approach to have potential in the community, an ultrasimple, portable, rugged system is required. We have recently suggested a design for a miniature fluorimeter based on LED excitation and a small, rugged spectrometer.^24^ We acknowledge that there are many such designs reported. Figure 2 shows an implemented design that is battery-operated and small. Briefly, the design leverages recent advances in very bright UV LEDs (∼50 −100 mW) and exceptionally small, high-resolution spectrometers (dimensions: 4, 4, 2.5 cm). The design uses 12 LEDs that span the UV-A,B, and C ranges, limited only by the commercial availability of LEDs.

**Figure 2:**
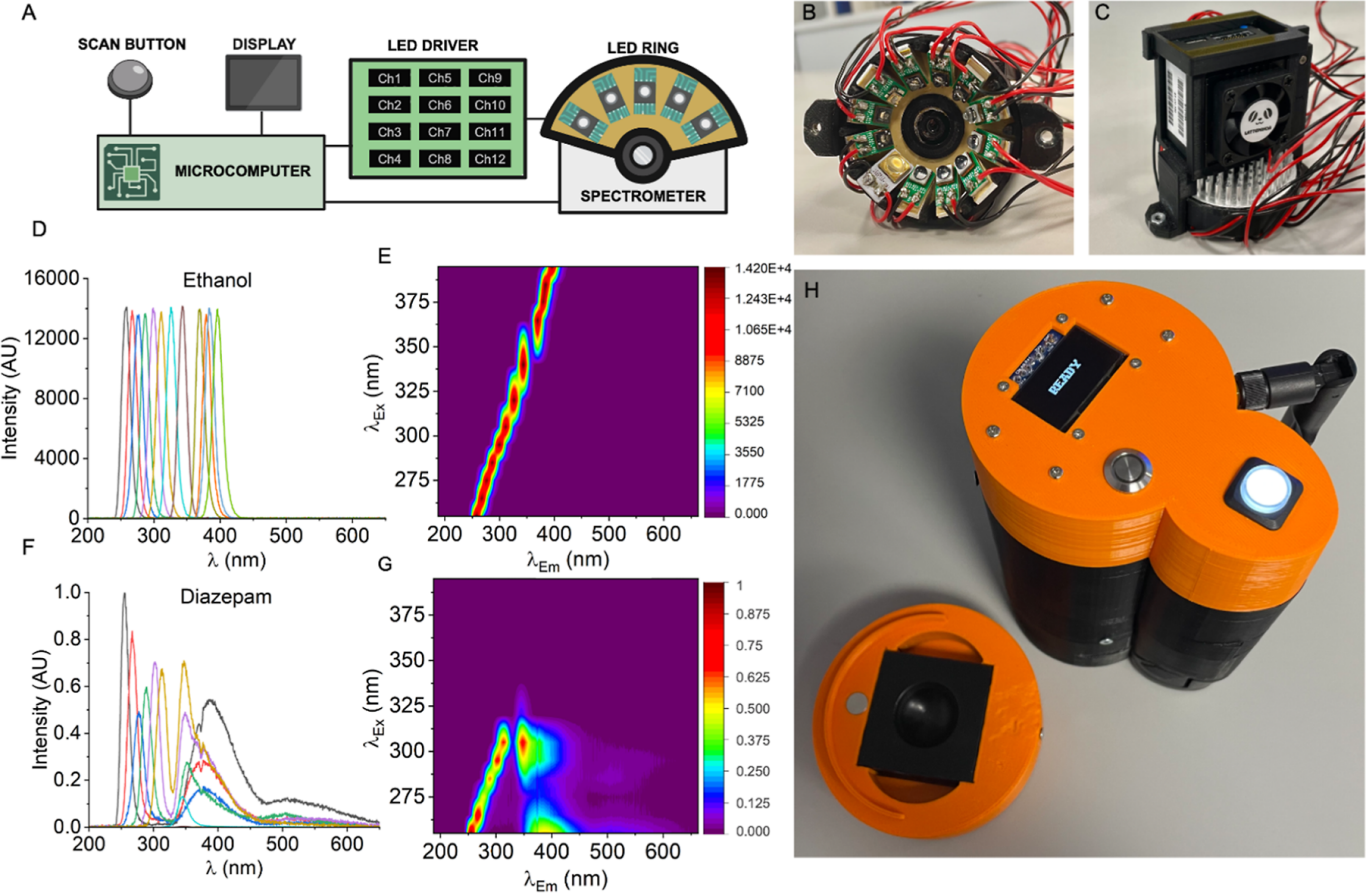
(A) Schematic of device architecture. (B) LED ring, where 12 LEDs are in a 1-in. diameter ring arrangement, equally spaced. (C) Spectrometer in a custom housing on the LED ring. Note the presence of a collimating lens in the front of the spectrometer. The entrance of the collimating lens is 15 mm from the sample surface. (D) 1D spectra of 2 mL ethanol recorded with the device, showing the reflected LED light. Emission (λ_em_) captured between 185 and 655 nm with excitation (λ_ex_) performed by 12 LEDs between 255 and 400 nm. These data are not averaged and have not been normalized. (E) Data in panel D shown as a contour plot. (F) 1D spectra of 2 mL 0.5 mg/mL diazepam standard in the sample holder. Normalized for integration time by channel and then 0–1 normalized. These data are not averaged. (G) Data in panel F shown as a contour plot. The color bar shown here is representative of all FSFs shown below. Corresponding unprocessed spectra are shown in Figure S1. (H) Complete prototype device showing both multifunctional buttons and the display. Note the liquid sample holder is composed of black HDPE, with a custom machined well (1 in. Wide, 10 mm deep, and of a true hemispherical construction). Note the device is shown in operation in Movie S1. Figure created in BioRender.

In our design, the analyte is dissolved in EtOH. As we discuss below, this enables concentration discrimination versus % purity, which we argue is the more useful information to give to a person intending on using the drug. The solubilized sample is irradiated in a custom holder composed of a UV- and chemically resistant material that is simple to wipe clean and holds a 2.2 mL volume. The total cost of the device, as shown in Figure 2 is ∼$2,000, with the cost being dominated by the spectrometer. To analyze a sample using the device, we have developed a standard operating procedure (SOP) where a sample must be crushed, incubated in 2.5 mL ethanol for 2 min, and then filtered before 2 mL of this solution is transferred to the sample holder.

The device benefits from being operated by a customizable microcomputer, meaning that both data acquisition and analysis can be controlled. The device operates by optimizing the signal such that the detector integration time is consecutively increased until the detector is at 80% saturation, assuming a linear relationship between the signal size and integration time, which is a reasonable approximation. Figure 2D,E shows the resultant output of the device with EtOH present in the sample holder, both as a one-dimensional spectrum (Figure 2D) and a contour plot (Figure 2E). The data essentially show the specular- and diffuse-reflection from the excitation sources since there is no analyte present, and we note these data are not normalized for differences in integration time. For clarity, in fluorescence spectroscopy (e.g., Figure 1), the reflected excitation source data are almost always excluded since they typically dwarf emission signals. However, as we discuss below, we have found these data to be useful, and so the geometry of the device shown in Figure 2 is set up to achieve some approximate parity between these signal sizes (acknowledging this varies widely for different molecules) and fluorescence emission (discussed below). Note that the contour plot graphic shown in Figure 2E interpolates between the data points to give the illusion of a higher resolution scan and is to aid the eye only.

Figure 2F,G shows the collected data with Diazepam at a relatively low concentration (0.5 mg/mL) compared to the pharmaceutical dose (we consider the concentration effect in detail below). From these data, there is clear discrimination of the emission resulting from the analyte, manifesting as new emission bands centered largely at ∼λ_em_ = 355 nm. We note that LEDs >305 nm are not visable to the eye due to normalization.

### Hybridized Fluorescence and Reflectance Fingerprints

With our device in hand, we consider how it can be implemented to discriminate between the BZD type and concentration. The magnitude of fluorescence emission is related to the concentration of a fluorophore; however, the magnitude of emission is usually a poor means to assess concentration, owing to a raft of potential convolving effects including collisional quenching, FRET, and the inner filter effect. Figure S2 demonstrates this challenge for a range of concentrations of diazepam, showing that the peak of the emission saturates with increasing concentrations (∼2 × pharmaceutical dose). These data illustrate that fluorescence emission alone becomes unreliable at high concentrations of an analyte. Instead, the absorption of an analyte is a more reproducible means to assess the concentration.

Absorbance spectra were measured for 24 benzodiazepine standards, and these data are shown in Figure S3. As expected, a significant variation in absorbance across different BZDs was observed, consistent with the variation in the electronic structure. Consistent absorption features between compounds of the same structural class (Figure S3) were also observed. While the variation in electronic absorption spectra with variation in structure is not a surprise result, it demonstrates the principle that different BZDs will absorb different wavelengths of light preferentially and characteristically. As such, we suggest that hybridizing both the FSF and information from analyte absorption might provide a means to both identify and quantify benzodiazepines, which we refer to as hybrid spectral fingerprinting (HSF).

Potentially, the magnitude of the reflected excitation source contains a wealth of information about the absorption of an analyte. That is, as the concentration of a fluorophore increases, the magnitude of the signal from the reflected excitation source decreases owing to an increase in absorption. Clearly, the closer in wavelength the excitation source is to the absorption maximum, the more pronounced is this effect. While not a “clean” absorption measurement like those shown in Figure S3, the reflected LED light represents a “pseudoabsorption” measurement, containing information on the absorption of the analyte at the LED wavelengths used for excitation. Indeed, so-called diffuse reflectance spectroscopy is used very commonly for the analysis of opaque samples.^28^

We have considered whether the reflectance data provide useful information on analyte concentration. Figure 3 shows a comparison between structurally different BZDs, showing their absorption spectra and HSFs. The HSFs are reported at three different concentrations: a “low”, “medium”, and “high” concentration relative to the pharmaceutical dose, respectively. In all three examples, the basic structure of the fingerprint is consistent with similar excitation emission maxima but shows variance that is obvious even to the eye as the concentration changes. These changes are apparent in both the emitted light (intensity and wavelength variation) as well as in the reflected excitation light (intensity variation). These data suggest that combining both the information from LED reflectance and fluorescence emission can not only identify different BZDs but also report on variation in BZD concentration. When using the device, the smallest difference in concentration that can be effectively distinguished is 0.1 and 0.2 mg/mL (from 0.25 and 0.5 mg alprazolam tablets).

**Figure 3:**
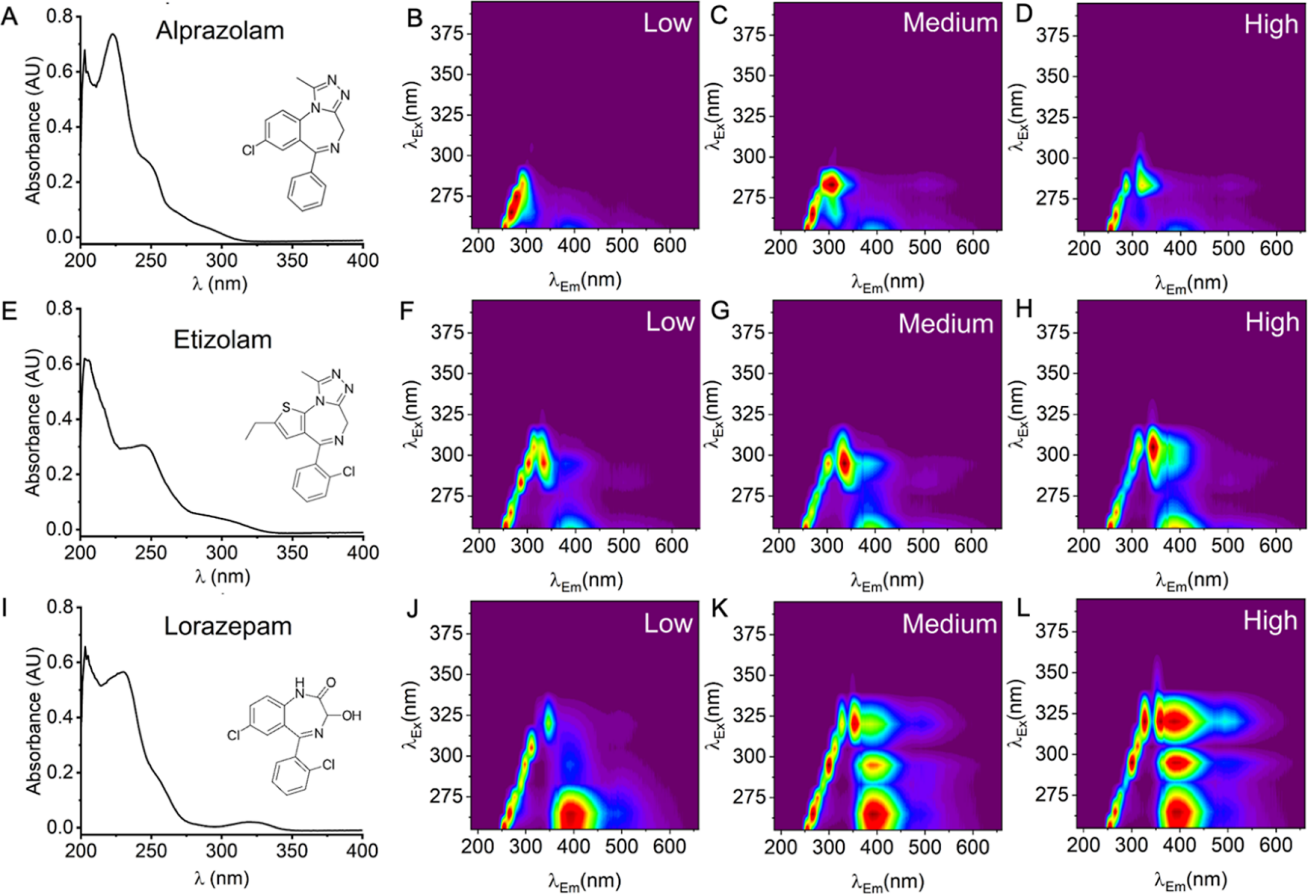
Relationship between absorption spectra, concentration, reflectance, and emission. (A–D) Alprazolam, low dose 0.25 mg (in 2.5 mL, 0.1 mg/mL), medium dose 0.5 mg (0.2 mg/mL), high dose 1.5 mg (0.6 mg/mL), (E–H) Etizolam, low dose 0.75 mg (in 2.5 mL, 0.3 mg/mL), medium dose 1.5 mg (0.6 mg/mL), high dose 3 mg (1.2 mg/mL), and (I–L) Lorazepam, low dose 0.5 mg (in 2.5 mL, 0.2 mg/mL), medium dose 2 mg (0.8 mg/mL), high dose 4 mg (1.6 mg/mL).

### Deep Learning Enables High Accuracy Discrimination of Both BZD Identity and Concentration

Ultimately, while the general principle of assessing concentration from a hybridized emission and pseudoabsorption measurement is feasible, we do not find an obvious generalizable model that allows extrapolation to different concentrations. For example, the inner filter effect will manifest differently for different analytes, and different analytes will have varying extinction coefficients. That is, the exact relationship is highly multivariate, making defining global rules for concentration measurement challenging, though we do not say impossible. Instead, we have explored whether deep learning can be deployed not only to identify BZDs from the fingerprints but also through exploiting the variants of drugs as well as their concentrations. In this way, we can enable rule generation by scanning a range of concentrations for each BZD into a data library. Our device makes this technically tractable since data are collected via high-intensity LEDs, with optimized spectrometer positioning, resulting in full fingerprint scans that are collected in ∼1 min, enabling very high sampling volumes.

For data collection, we captured HSFs for 86 distinct drug-variant-concentration classes, as shown in Table S1. Of these classes, 11 are BZDs at a range of concentrations that relate to relevant harm reduction advice: low, medium, and high [relative to pharmaceutical dose or as harm reduction information from community forums][Bibr ref29] and a range of other relevant drugs in combination. We acquired 20 HSFs for each condition where possible. However, for robustness, it was important to have a model that could provide accurate predictions for drugs even if very few samples were available for it because this is expected to occur for many real-world drug samples.

Raw data from each scan produces 18,192 rows of data across 5 features, namely, (1) the LED currently active, corresponding to the 12 LEDs that the device has and, therefore, represents the current excitation wavelength; (2) an emission wavelength being recorded by the spectrometer; (3) the light intensity at the given emission wavelength; (4) the integration time; and (5) the brightness of the LED, in the range [0, 1], which is adjusted using pulse-width modulation (PWM) to reduce excitation to levels below what the LEDs are capable of.

Data from device scans comprise the data set used for training predictive models (Table S1) and are each manually labeled with drug, variant, and concentration information. Before being used in training predictive models, the dataset was preprocessed as follows: (i) nonspectral data, such as excitation wavelengths and integration times, were removed; (ii) spectral data were trimmed to only the range of wavelengths containing useful fluorescence information; (iii) spectral data of similar wavelengths were averaged together to reduce its dimensionality; (iv) spectral data from each excitation wavelength were normalized to the range [0,1] to improve computational efficiency; and (v) data were padded and reshaped into a 66 × 66 × 1 matrix.

The preprocessed data are passed to a convolutional neural network (CNN) deep learning model, which uses the custom architecture shown in Figure 4A. With this architecture, each HSF is passed into the model’s input layer, which is then processed through multiple hidden layers, namely, (i) three occurrences of a convolution layer, which uses the rectified linear unit (ReLU) activation function and max pooling; (ii) data were then flattened to be fed into fully connected dense layers, akin to a standard neural network, which also uses the ReLU activation function; and (iii) the dense layers led to a final output layer, which uses a Softmax activation function to produce probabilities for each of the 86 classes listed in Table S1.

**Figure 4:**
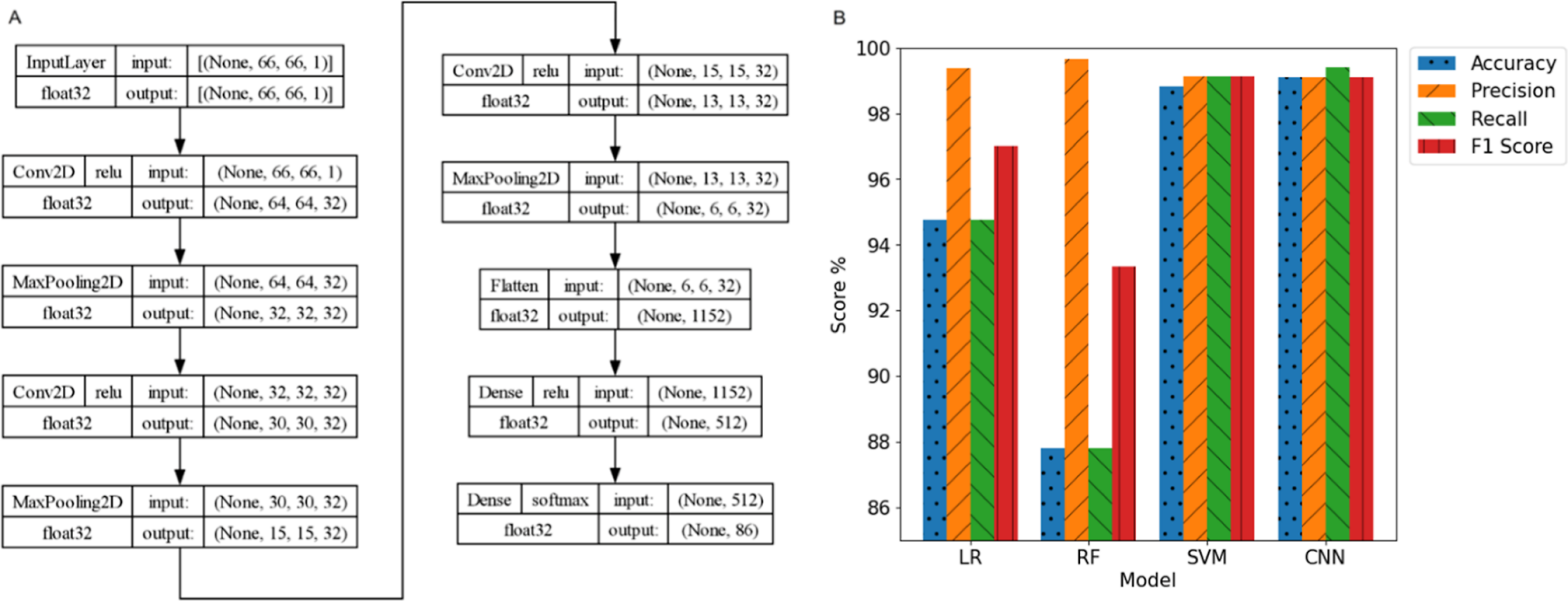
(A) Architecture of the CNN model, depicting its initial input layer, which corresponds to preprocessed spectral data that is padded and reshaped into a 66 × 66 × 1 matrix, followed by hidden layers of convolution and pooling, and dense layers with rectified linear unit (ReLU) and softmax activation functions, which result in probabilities for each of the 86 drug classes listed in Table S1. (B) Accuracy, precision, recall, and F1 scores of the candidate algorithms used to train potential models for the device: logistic regression (LR), random forest (RF), support vector machine (SVM), and CNN. The CNN model yielded the highest accuracy and was chosen as the model to deploy onto the devices.

The data set was established using the device itself by scanning known substances, as well as various common nondrug classes to aid model robustness, for example, caffeine, paracetamol, and white paper. In total, the data set contains 1470 HSF examples. Most classes have 20 examples each, and those with fewer than 20 had their data oversampled, with replacement, to balance the data set and reduce model bias. This results in a total of 1720 HSF examples used by models: 20 examples for each of the 86 classes. The 1720 examples were preprocessed, randomly shuffled, and split so that 80% (1376) were used for model training and 20% (344) for testing.

To ensure that a sophisticated deep learning model, such as CNN, was the most appropriate choice of model for this task despite its larger computational overhead, three standard machine learning (ML) models were also trained with the same data set, namely, LR, RF, and SVM. Adjustments were made so that models from these three algorithms could function in a comparable manner to CNN, as follows: (i) each model was configured to be a One-vs-Rest (OvR) classifier so that they could provide multiclass classification; (ii) a large hyperparameter search was conducted for each model to determine the optimal model configuration, for example, by trying a range of regularization strengths {0.0, 0.2, 0.4, 0.6, 0.8, 1.0} and kernel functions {linear, polynomial, radial basis function, sigmoid}; and (iii) a set of PCA values {25, 50, 75, 100} was used during the hyperparameter search so that dimensionality reduction could be included in model design, comparable to the use of max pooling in the CNN architecture. The preprocessing used for CNN data was also used for the data used by the ML models, resulting in the same feature set and data dimensions for all models.

After training models for all four architectures, the models with the highest accuracy per algorithm are shown in Figure 4B, alongside their respective scores for the Precision, Recall, and F1 Score metrics. The results show that the CNN model has the highest accuracy overall at 99.1%, followed by SVM at 98.8%, RF at 87.8%, and LR at 94.8%. The CNN model yielded the greatest accuracy overall and was chosen for deployment on the devices.

During testing, the CNN model made 3 incorrect predictions, namely, (i) it predicted flubromazolam of high concentration as flubromazolam of medium concentration once, and (ii) it predicted ketamine from a specific sample with ketamine from a different sample twice. It is encouraging that these misclassifications are still correctly classifying the right drug even if the variant and concentration are incorrect. Although SVM is similar in accuracy to CNN, it confused two different drugs, namely, flubromazolam with 2C–B.

Taken together, by leveraging a hybridized fluorescence and pseudoabsorption measurement, implemented on an optimized device geometry, we can apply deep learning to discriminate not just BZD type but also variant and concentration information that is relevant for harm reduction information.

### Effect of Excipients and Mixtures on BZD Discrimination

The success of other field-deployable devices in identifying designer benzodiazepines is limited by their poor detection in samples of or prepared from tablet material. Lactose monohydrate and microcrystalline cellulose are the primary excipients used in pharmaceutical benzodiazepine tablets and are compounds previously reported as producing strong interfering signals in both Raman spectroscopy and FTIR instruments.^22,23^ Having already validated the effectiveness of resolving benzodiazepine standards via HSF, Figures S4 show the comparative absorbance spectra taken for four compounds (clonazepam, diazepam, nitrazepam, and zopiclone) when extracted from pharmaceutical tablets. We find that spectra are highly reproducible across tablet extracts and standard solutions, where no additional peaks are present, and all absorbance maxima are preserved. From Figure S4, the HSFs are essentially identical, whether from a standard or extracted from a tablet. That is, the approach is not affected by the presence of typical excipients, and they do not contribute to the measured HSFs.

Unlike licensed medication, street BZDs are manufactured with little regard for quality control and are frequently cut with over-the-counter painkillers, including paracetamol and ibuprofen, in addition to stimulants such as caffeine.^30^ Many of these adulterants contain conjugated ring structures that are known to exhibit intrinsic fluorescence;^31,32^ therefore, we sought to examine the potential impact of these compounds on benzodiazepine FSF.

Figure 5 shows the resultant FSFs of 1 mg/mL diazepam tablet extract combined with caffeine, paracetamol, and ibuprofen. The ratio of cutting agents to benzodiazepine is typical of what could be expected for a street tablet (∼20:1), excluding caffeine, which was added at a lower concentration owing to solubility in EtOH, recapitulating realistic extraction from street tablet material.^30^ Diazepam fluorescence is readily resolved across these samples where emission contributions from caffeine (λ_ex_ 265, λ_em_ = 310 nm), paracetamol (λ_ex_ 285, λ_em_ = 325 nm), and ibuprofen (λ_ex_ 265, 275, 285 nm, λ_em_ = 290 nm) are also partially preserved. In Figure 5E,I, we show how the spectral features of clobazam are also resolved in the presence of diazepam.

**Figure 5:**
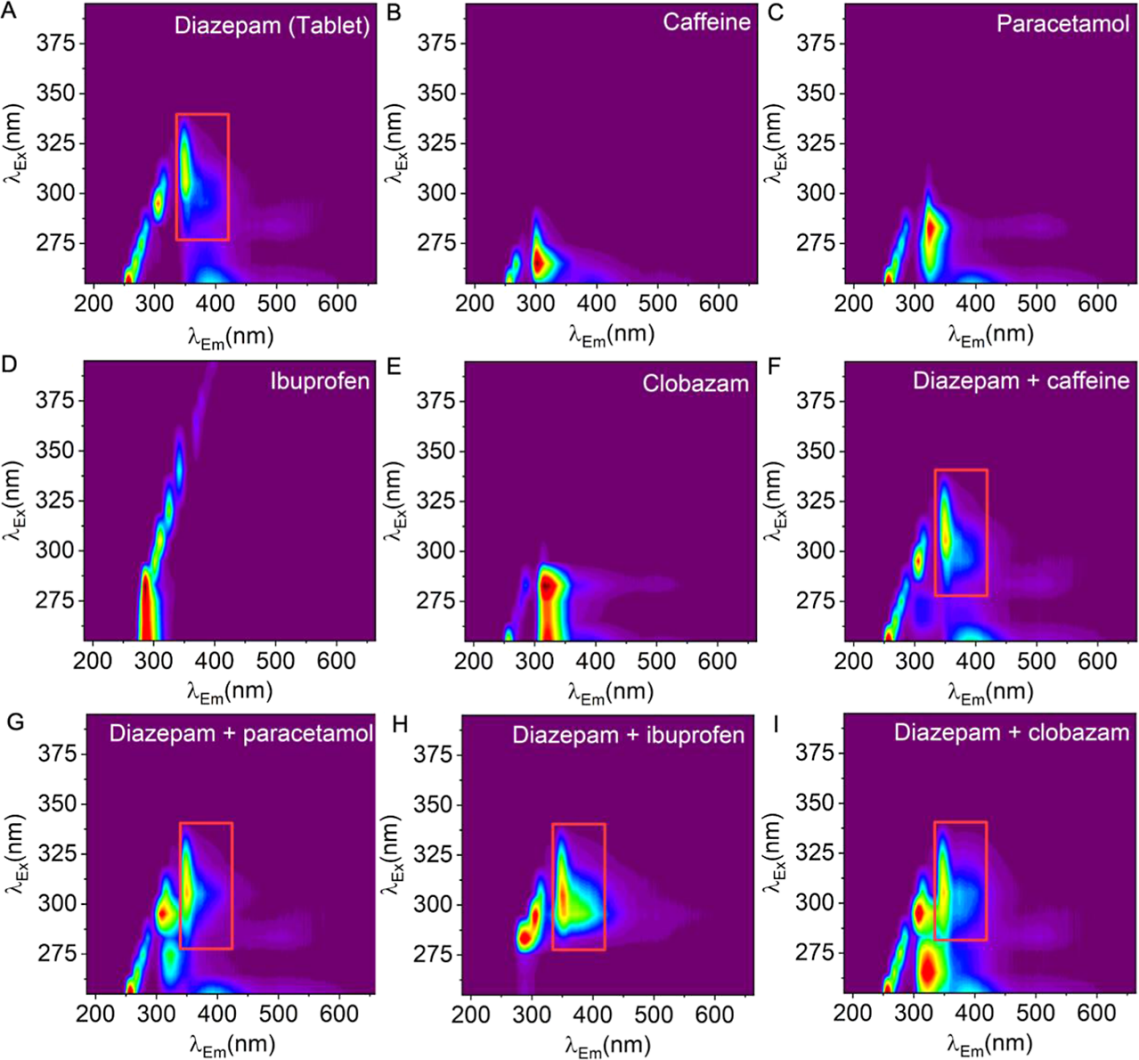
Diazepam samples prepared from pharmaceutical tablet material in the presence of common cutting agents and other benzodiazepines. (A) Diazepam 1 mg/mL tablet extract, (B) caffeine 0.25 mg/mL, (C) 20 mg/mL paracetamol, (D) 20 mg/mL ibuprofen, (E) 1 mg/mL clobazam tablet extract, (F) diazepam and caffeine (concentrations preserved), (G) diazepam and paracetamol, (H) diazepam and ibuprofen, and (I) diazepam and clobazam.

These data highlight that spectral features arising from the presence of multiple fluorescent species within a scanned sample are generally additive in nature. We therefore posit that positive identification of multiple benzodiazepines might be achieved in a single sample of tablet material through the training of a CNN predictive model that incorporates examples of various compound combinations. However, we note that while the fluorescence emission in Figure 5 is essentially additive with additional emissive compounds, the reflected LED light varies in a more complex manner. Moreover, we do not anticipate that it will always be the case that emission spectra will be additive, so we advocate for careful analysis of known contaminants and likely or observed mixtures.

### Discrimination of Complex Drug Mixtures Beyond BZDs

Clearly, there are other potential drugs of abuse that could be detected by using this approach. Figure 6 shows a range of HSF data for different molecules, including drugs that are topical because of their association with significant rates of overdose and death, including fentanyl, xylazine, heroin, and examples of nitazenes. From Figure 6, each of the fingerprints is trivial to identify, even by eye.

**Figure 6:**
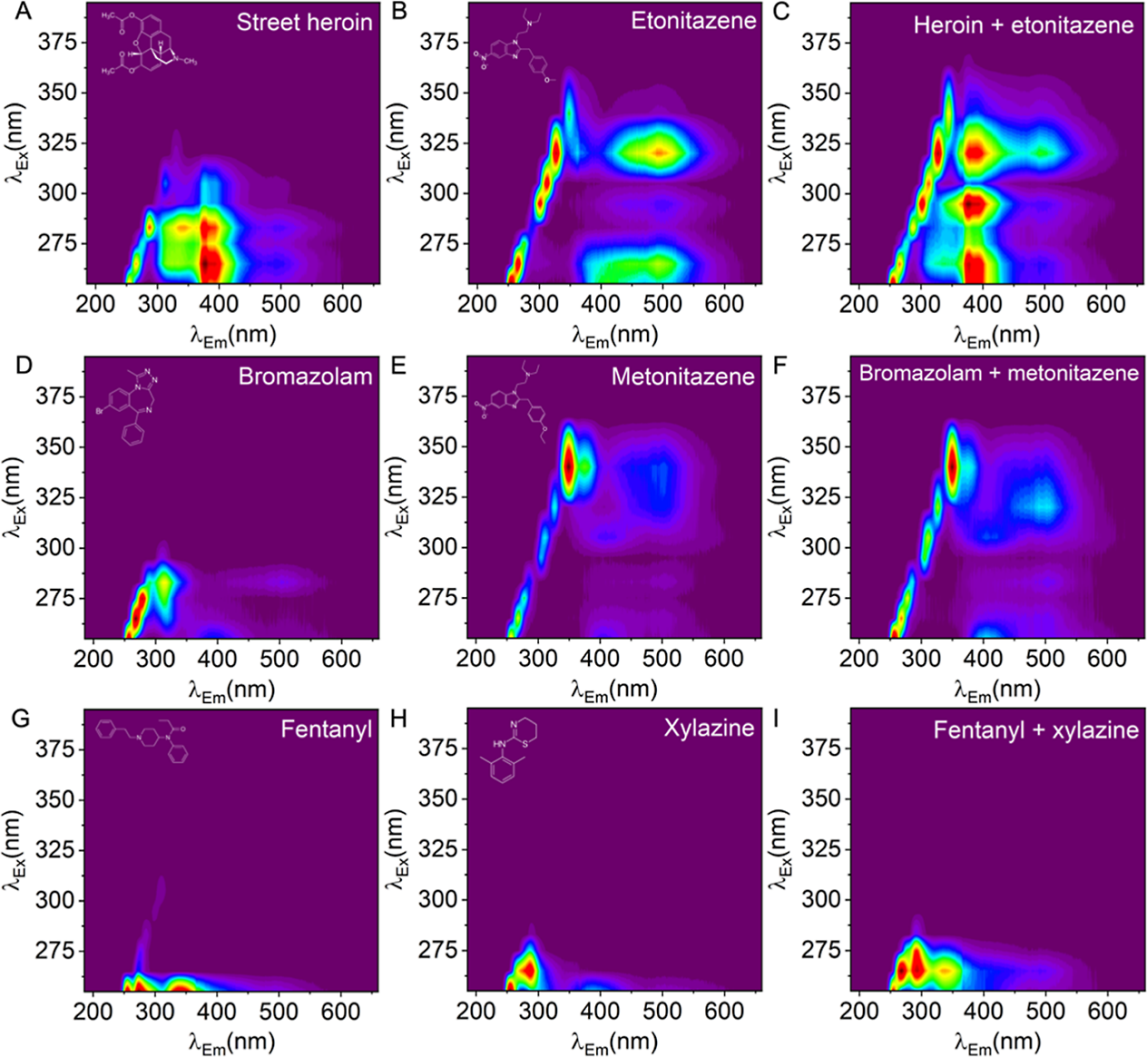
Detection of relevant mixtures of street drugs. (A) Street heroin containing heroin and noscapine, 1 mg/mL, (B) etonitazene, 0.5 mg/mL, (C) heroin and etonitazene, (D) bromazolam, 0.2 mg/mL (low dose), (E) metonitazene 0.5 mg/mL, (F) bromazolam and metonitazene (concentrations preserved), (G) fentanyl, 1 mg/mL, (H) xylazine 0.33 mg/mL, and (I) fentanyl and xylazine (concentrations preserved).

While these drugs individually are responsible for drug overdose, the combinations present a significantly enhanced risk of harm and death,^33,34^ so their discrimination is critical to useful harm reduction strategies that could employ the approach. Figure 6C,F,I shows the fingerprint of the combined drug mixture from the panels at left. Similar to our findings in Figure 5, the HSFs are essentially additive with the key spectral features of each drug being retained. We note that the main peak for bromazolam (λ_Ex_ = 283, λ_Em_ = ∼310 nm) is retained in the mixture but is difficult to observe by the eye in the contour plot shown in Figure 6F. Using our deep learning approach described above (Figure 4), we find that these drugs and mixtures are easily discriminated both from one another from the rest of the library of benzodiazepines.

## Conclusions

Our previous work discriminating synthetic cannabinoids using EEMs suggested the potential of discrimination of other drugs that are likely to be fluorescent.^24^ A survey of the structures of some of the key drugs of abuse (synthetic opioids and benzodiazepines and many others) suggests that fluorescence is likely to be observable in a great many cases owing to the prevalence of a conjugated ring system in many drugs, which then have the potential for relatively bright emission. There are many examples, including our own work with synthetic cannabinoids, which demonstrate that minor substitutions to conjugated ring systems and or to a network of cross-conjugated bonds are sufficient to alter the profile of excitation and emission and in a manner that is characteristic and reproducible for a given solvent.

Our data show that this is the case with a range of benzodiazepines, which we use as an example of a large class of structurally diverse illicit drugs, but it also extends similarly to opioids. We extend these measurements to include information from reflectance, which we find enables not only enhanced discrimination of the analyte but also supports concentration discrimination. To establish the generality of this principle, we show HSF data for key examples of illicit drugs in Figure 7A–I. These examples now include four additional opioids that are licensed medications in the UK (7A–D), common recreational “party drugs” (7E–G; cocaine, MDMA, ketamine), and two NPS not discussed in the main paper [7H,I; a synthetic cathinone (mephedrone) and a psychedelic (2C–B)]. Even to the eye, the HSFs are simple to discriminate and point to the more general utility of this tool. We remind readers that these data are collected on a field-portable device and so illustrate the very high potential for these measurements to support community harm reduction activities, including in outreach settings, without the need for specialist device users. We note the need to adhere to an SOP when analyzing samples using our device, and deviation from this will invalidate results. However, the SOP is simple to follow, requiring a single button press to operate the device and obtain a sample result. We acknowledge that some benchtop instruments (i.e., FT-IR) can also be effectively used in community drug checking services; however, these are still limited by lack of portability and cost and still generally require specialist knowledge for operation. Our device is further advantaged in these scenarios, as connection to a laptop/monitor is not required for interpretation of sample results.

**Figure 7:**
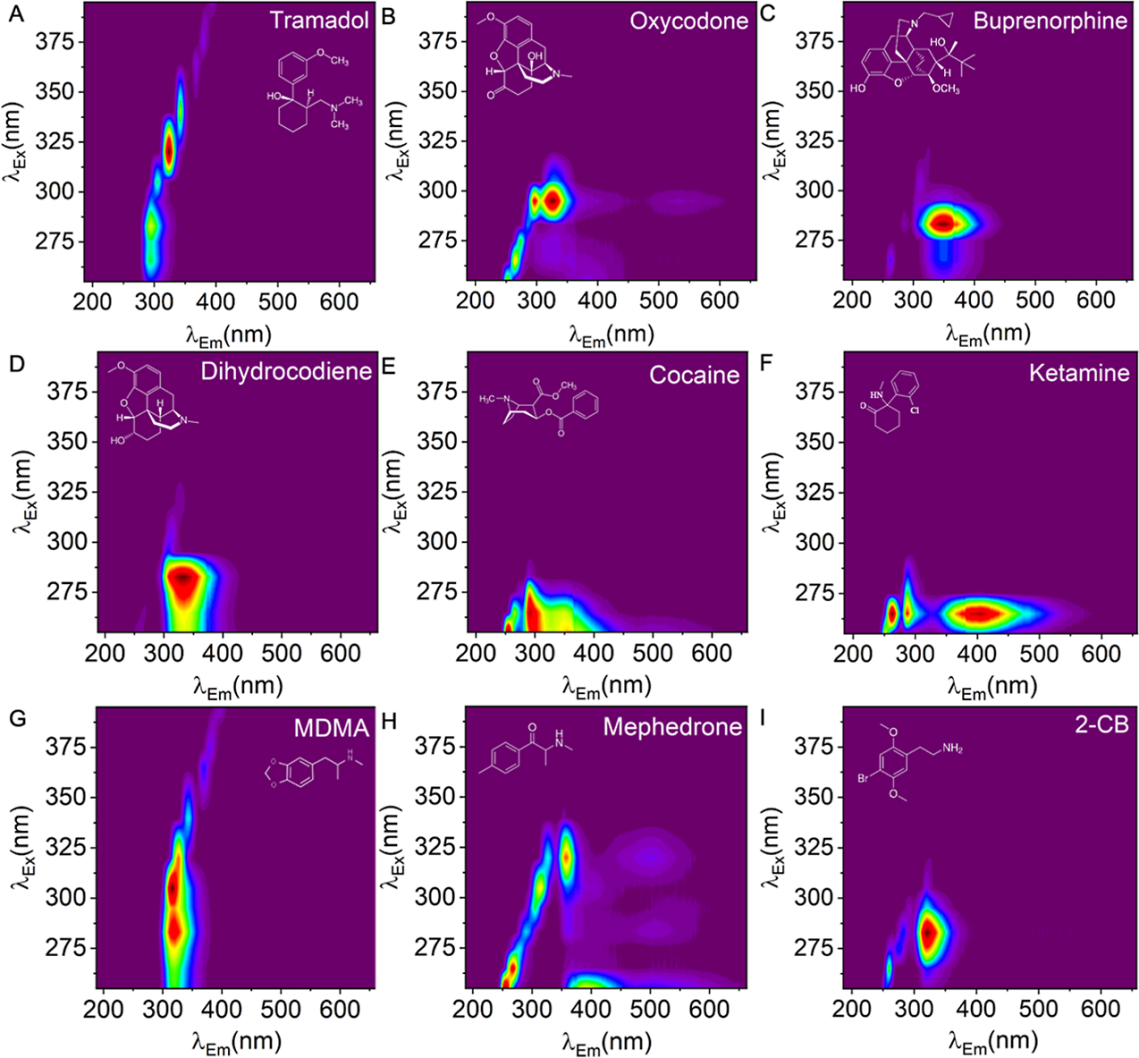
Detection of wider range of compounds, including a range of prescription opioids and club drugs. (A) tramadol 50 mg (20 mg/mL), (B) oxycodone 10 mg (4 mg/mL), (C) buprenorphine 2 mg (0.8 mg/mL), (D) dihydrocodeine 30 mg (12 mg/mL), (E) cocaine 2 mg/mL, (F) ketamine 2 mg/mL, (G) MDMA 2 mg/mL, (H) mephedrone 2 mg/mL, and (I) 2CB 2 mg/mL.

Our data illustrate that HSF data are highly discriminatory and can provide both identification and concentration information. The HSF data are highly complex, and we find that an implementation of discrimination by deep learning is effective. While we do not suggest this approach will ever rival lab-based analysis in terms of sensitivity or specificity, we suggest it can be a powerful tool for use by nonexperts in the field. We demonstrate the potential of this through a field-portable prototype that is small, robust, and inexpensive.

## Materials and Methods

### Device Design

Device design is outlined in the results text in addition to mission-critical AIA and ML algorithms. Specific components include an ST UV microspectrometer (Ocean Insight), a TMP36 temperature sensor (Analog Devices), a monochrome OLED display, and a triple-axis accelerometer (Adafruit). Bespoke Python software was loaded on a Raspberry Pi Zero 2 W microcomputer running the Raspberry Pi operating system on a 32GB MicroSD card. Twelve LEDs were driven at 350 mA using a custom PCB. Custom holder for 12-channel LED ring, heat sink, and ST spectrometer 3D-printed using an Ultimaker S3 printer using ABS plastic.

### Standard Solution and Tablet Sample Preparation

All standards were purchased as 1 mg/mL solutions in methanol (Merck/Cayman) and were diluted for absorption and fluorescence measurements in 99.9% HPLC-grade 2-propanol (VWR). Standards were stored at −20 °C according to the manufacturer’s recommendations. Drug material (including street benzodiazepine tablets and powders) was provided by police from seizures, TICTAC Communications Limited, and Manchester Drug Analysis & Knowledge Exchange (MANDRAKE). Pharmaceutical tablets were obtained from the School of Pharmacy at the University of Bath through a commercial supplier. Solutions were prepared from tablet material by crushing the whole tablet with a pestle and mortar. Powder was suspended in 2.5 mL ethanol, and material was shaken for 10 s then left to settle for 120 s. Samples were filtered to remove insoluble debris, and pelleted material was discarded.

### Fluorescence and Absorption Spectroscopy

Emission maps were acquired using a spectrofluorometer (Edinburgh Instruments, FS5) paired with a temperature-controlled cuvette holder (SC-25 TE Cooled-Standard) and a temperature controller (TC1, Quantum Northwest). Emission maps were captured at 20 °C with excitation scanned in 5 nm intervals between 260 and 400 nm and emission scanned in 0.5 nm intervals between 275 and 600 nm. Quartz fluorescence cuvettes were used to collect emission spectra for solutions of 1 mL sample volume. Data were background-subtracted to remove contributions from Raman scattering peaks and trimmed to remove the monochromator excitation peaks. Data were normalized and then plotted using Origin Pro.

Absorption measurements of benzodiazepine standards and tablet solutions were acquired using an Agilent Technologies Cary 60 UV–visible (UV–Vis) spectrophotometer. Temperature was maintained at 20 °C for all experiments using a Peltier. Quartz fluorescence cuvettes were used to collect absorbance spectra for solutions of 1 mL sample volume. Absorbance was recorded between 800 and 200 nm with a scan rate of 600 nm/min and 1 nm intervals between data points.

## Supplementary Material





## Notes

Device in operation (MP4)
HSF spectral variation with respect to concentration, illustrative absorption spectra, representative HSF spectra, and deep learning data (PDF)
