# Supplementary material for: Field-Portable Technology for Illicit Drug Discrimination via Deep Learning of Hybridized Reflectance/Fluorescence Spectroscopic Fingerprints
Source: Anal Chem. 2025 May 7;97(19):10163–72. doi: 10.1021/acs.analchem.4c05247 (PMC12096345; doi:10.1021/acs.analchem.4c05247)
Supplement: Supplementary file 2 [file ac4c05247_si_002.pdf]

Supporting Information. A field-portable technology for illicit drug discrimination via deep learning of hybridized reflectance/fluorescence spectroscopic fingerprints

Alexander Power,[1‡] Matthew Gardner,[2‡] Rachael Andrews,[2] Gyles Cozier,[2] Ranjeet Kumar,[2] Tom P. Freeman,[3] Ian S. Blagbrough,[2] Peter Sunderland,[2] Jenny Scott,[4] Anca Frinculescu,[5] Trevor Shine,[5] Gillian Taylor,[6] Caitlyn Norman,[7] Hervé Ménard,[7] Niamh N Daéid,[7] Oliver B. Sutcliffe,[8] Stephen M. Husbands,[2] Richard W. Bowman,[9*] Tom S. F. Haines,[1*] Christopher R. Pudney[2*]

[1]Department of Computer Science, University of Bath, Bath BA2 7AY, UK

[2]Department of Life Sciences, University of Bath, Bath BA2 7AY, UK

[3]Department of Psychology, University of Bath, Bath BA2 7AY, UK

[4]Centre for Academic Primary Care, Bristol Medical School, University of Bristol, Bristol, BS8 2PS, UK

[5]TICTAC Communications Ltd., Room 1.159 Jenner Wing, St. George's University of London, Cranmer Terrace, London SW17 0RE, UK

[6]School of Health and Life Sciences, Teesside University, Middlesbrough, TS1 3BX

[7]Leverhulme Research Centre for Forensic Science, University of Dundee, Dundee, DD1 4HN

[8]MANchester DRug Analysis & Knowledge Exchange (MANDRAKE), Department of Natural Sciences, Manchester Metropolitan University, Manchester, M1 5GD

[9]School of Physics and Astronomy, University of Glasgow, Glasgow, G12 8QQ, UK

[10]Centre for Therapeutic Innovation, University of Bath, Bath BA2 7AY, UK

Corresponding Authors * richard.bowman@glasgow.ac.uk, tsfh20@bath.ac.uk, c.r.pudney@bath.ac.uk

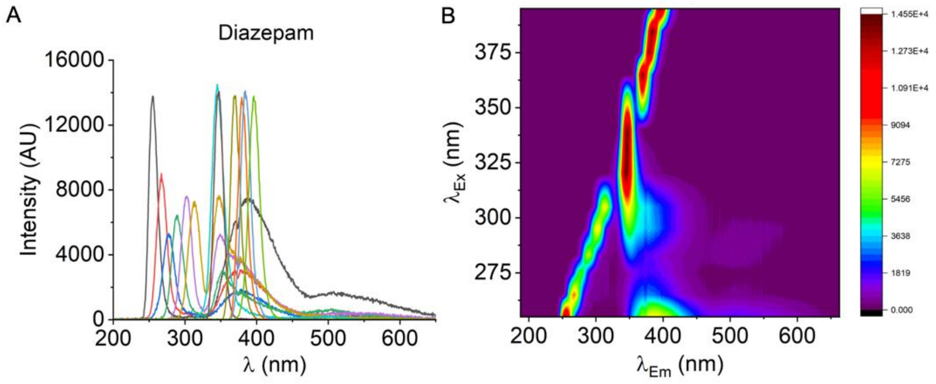

Figure S1. Unprocessed spectra corresponding to Figure 2 (F, G). (A), 1D spectra of 2 mL 0.5 mg/mL diazepam standard recorded by the device. These data are not averaged or normalized. (B), Contour plot of A.

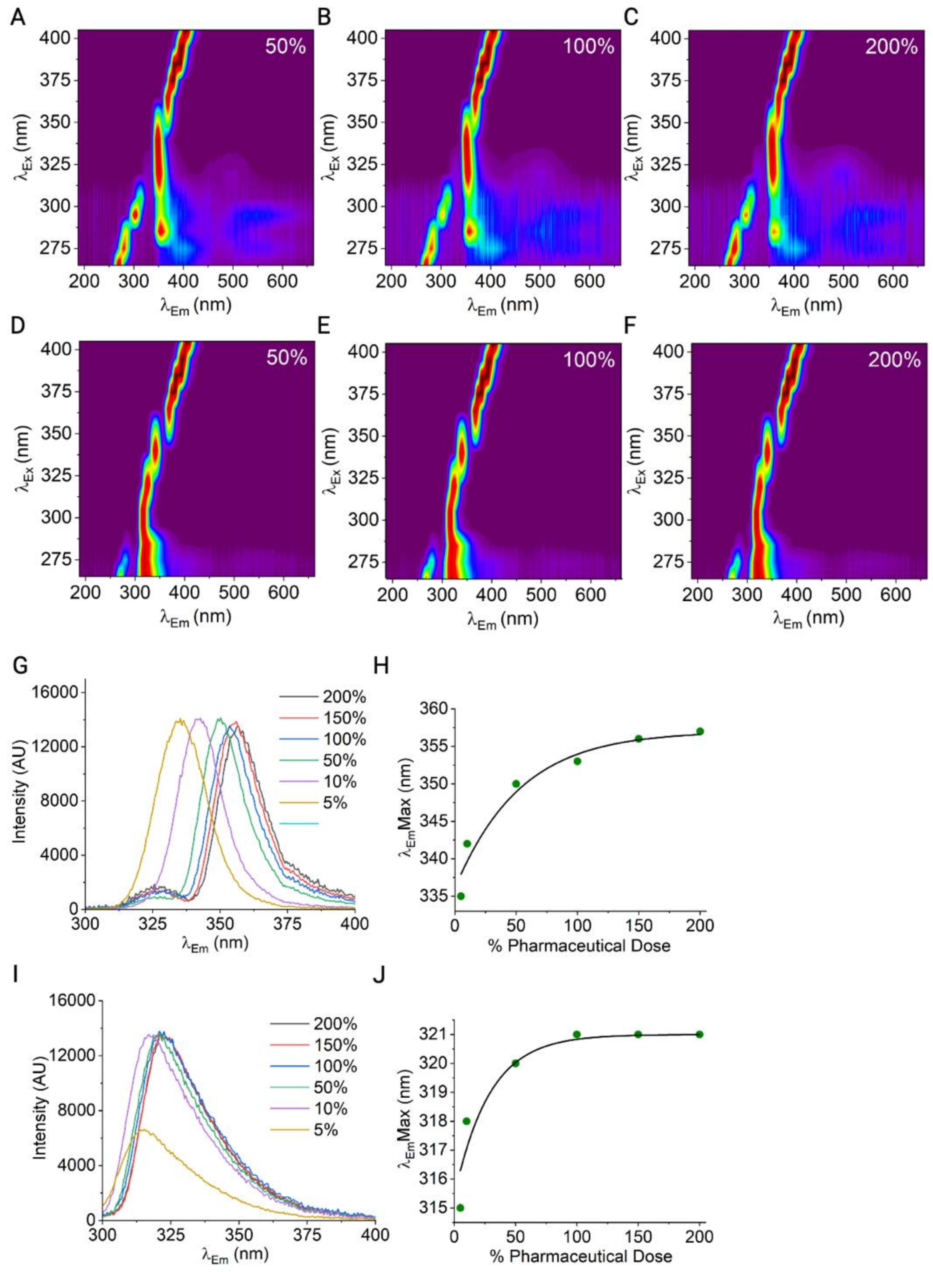

Figure S2. HSFs for diazepam and clobazam at a range of concentrations expressed as percentage of pharmaceutical dose, where 5mg/mL is equivalent to 100 % for both. (A-C), FSFs for 50 %, 100 % and 200 % diazepam pharmaceutical tablet extract. (D-F), FSFs for 50 %, 100 % and 200 % clobazam pharmaceutical tablet extract. (G), Shift in emission max for diazepam from 5 % to 200 % pharmaceutical dose (0.25-10 mg/mL). (H), Plotted relationship of diazepam fluorescence emission peak (channel 6) against concentration. (I), Shift in emission max for clobazam from 5 % to 200 % pharmaceutical dose (0.25-10 mg/mL). (J), Fluorescence emission peak of clobazam (channel 4) plotted against concentration.

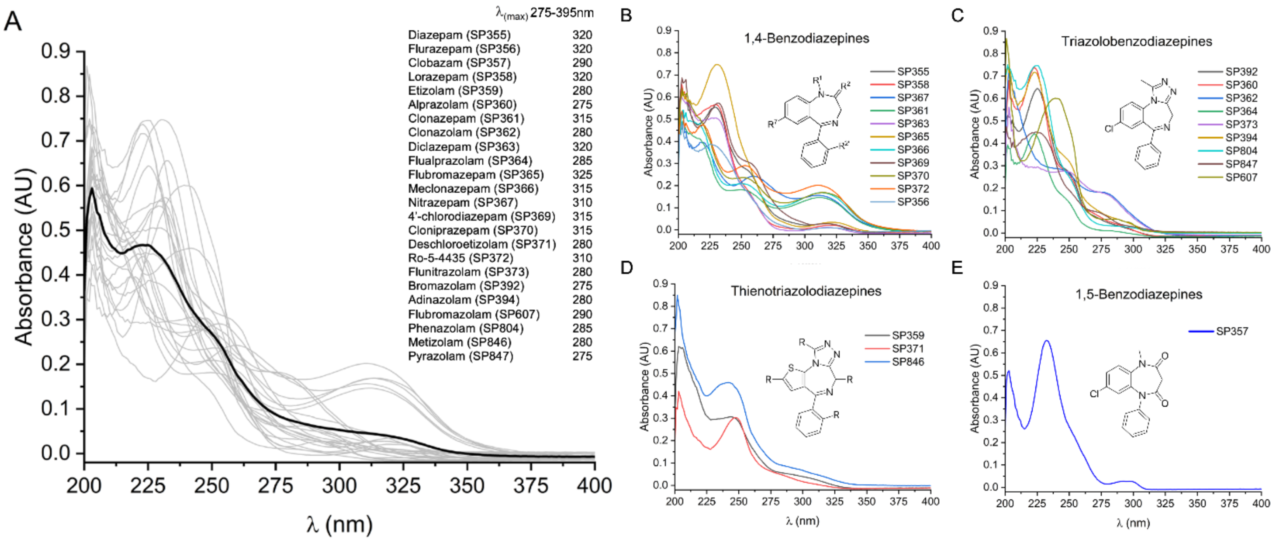

Figure S3. Absorbance spectra for a selection of 24 benzodiazepine standard solutions, 5 µg/mL. (A), Overlay of spectra for all compounds (grey) with mean average (black). (B), Grouped spectra for the 1,4-benodiazepines with exemplar chemical structure. (C), Grouped spectra for the triazolobenzodiazepines. (D), Grouped spectra for thienotriazolodiazepines. (E), Absorbance spectrum of clobazam, a 1,5-benzodiazepine.

Table S1. The dataset used for training predictive models. The class names of the training examples are derived from the Drug, Variant, and Concentration labels below, for example: "Heroin [SP908, 1mg-mL]". Labels that are not applicable are listed as "NA" and are excluded from the class name, for example: "No Drug [White Paper]".

Drug	Variant	Concentration	# Samples
2C-B	NA	2mg/mL	14
4-AcO-DMT	NA	NA	13
4-MMC	SP940	2mg/mL	20
Alprazolam	NA	Low (0.25 mg: 0.1 mg/mL)	14
		Medium (0.5 mg: 0.2 mg/mL)	13
		High (1.5 mg: 0.6 mg/mL)	20
Amphetamine	SP956	2mg/mL	20
Bromazolam	BDP, SP993	NA	13
	BDP-2, SP995	NA	13
	TICTAC	0.15 mg: 0.06 mg/mL	13
	NA	Low (0.5 mg: 0.2 mg/mL))	20
		Medium (1 mg: 0.4 mg/mL)	20
		High (2 mg: 0.8 mg/mL)	20
Buprenorphine	NA	1mg/mL	14
Clonazepam	NA	Low (0.5 mg: 0.2 mg/mL)	13
		Medium (1 mg: 0.4 mg/mL)	20
		High (2 mg: 0.8 mg/mL)	20
Cocaine	SP909	1mg/mL	20
	SP938	2mg/mL	20
Diamorphine	NA	1mg/mL	14
Diazepam	NA	Low (5 mg: 2 mg/mL)	13
		Medium (10 mg: 4 mg/mL)	13
		High (20 mg: 8 mg/mL)	20
Etizolam	TICTAC, SP466	NA	13
	NA	Low (0.75 mg: 0.3 mg/mL)	20
		Medium (1.5 mg: 0.6 mg/mL)	20
		High (3 mg: 1.2 mg/mL)	20
Etonitazene-(FB)	NA	0.05mg/mL	20
		0.1mg/mL	20
		0.25mg/mL	20
		0.5mg/mL	20
Etonitazene-(S)	NA	0.05mg/mL	20
		0.1mg/mL	20
		0.25mg/mL	20
		0.5mg/mL	20
Flualprazolam	NA	Low (0.15 mg: 0.06 mg/mL)	20
		Medium (0.5 mg: 0.2 mg/mL)	20
		High (1.5 mg: 0.6 mg/mL)	20

Flubromazepam	NA	Low (2 mg: 0.8 mg/mL)	20
		Medium (5 mg: 2 mg/mL)	20
		High (10 mg: 4 mg/mL)	20
Flubromazolam	NA	High (0.5 mg: 0.2 mg/mL)	20
Heroin	SP1029	1mg/mL	14
	SP908	1mg/mL	20
	SP975	1mg/mL	14
	SP978	1mg/mL	14
	SP991	1mg/mL	14
Heroin+Nitazene	BU-20%-1	1mg/mL	14
	BU-20%-2	1mg/mL	14
	BU-20%-3	1mg/mL	12
	BU-20%-4	1mg/mL	20
	SP1019+SP986	1mg/mL	13
	SP1035	1mg/mL	12
	SP986	1mg/mL	13
Ketamine	SP932	2mg/mL	20
	NA	2mg/mL	11
LSD	NA	NA	10
Lorazepam	NA	Low-(H) (1 mg: 0.4 mg/mL)	20
		Low (0.5 mg: 0.2 mg/mL)	20
		Medium (2 mg: 0.8 mg/mL)	20
		High (4 mg: 1.6 mg/mL)	20
MDMA	NA	2mg/mL	10
Methamphetamine	NA	2mg/mL	10
Methylphenidate	NA	2 mg/mL	14
Metonitazene-(S)	NA	0.05mg/mL	20
		0.1mg/mL	20
		0.25mg/mL	20
		0.5mg/mL	20
Nitrazepam	NA	Low (5 mg: 2 mg/mL)	20
		Medium (10 mg: 4 mg/mL)	20
		High (20 mg: 8 mg/mL)	20
No Drug	Caffeine	0.5 mg/mL	20
	EtOH	NA	20
	Ibuprofen	80 mg/mL	14
	Paracetamol	200 mg/mL	14
	White Paper	NA	20
Oxycodone	NA	4 mg/mL	9
Temazepam	NA	Low (10 mg: 4 mg/mL)	13
		Medium (20 mg: 8 mg/mL)	20
		High (40 mg: 16 mg/mL)	20
Tramadol	NA	20 mg/mL	14
Xylazine	NA	1mg/mL	14
Xylazine+Caffeine	NA	2mg/mL	10
Zopiclone	NA	Low (3.75 mg: 1.5 mg/mL)	20
		Medium (7 mg; 2.8 mg/mL)	20
		High (15 mg: 6 mg/mL)	20

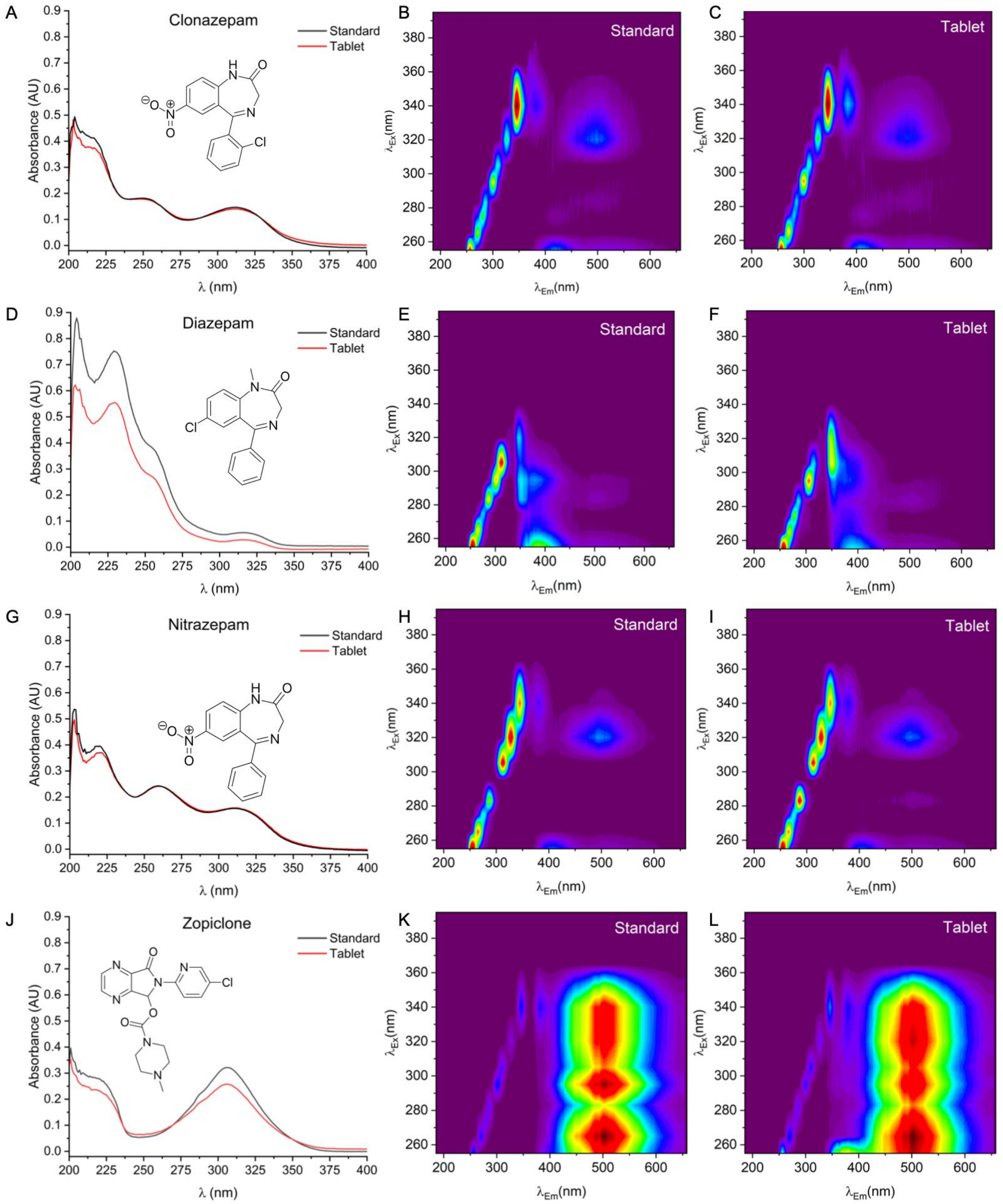

Figure S4. Comparative absorbance spectra and HSF for benzodiazepine standards and samples prepared from tablet material. (A), Absorption spectra for clonazepam standard and tablet extraction at 5 μg/mL. (B, C), HSFs for standard and tablet extraction at 0.4 mg/mL.(D), Absorption spectra for diazepam standard and tablet extraction at 5 μg/mL. (E, F), HSFs for standard and tablet extraction at 1 mg/mL. (G) Absorption spectra for nitrazepam standard and tablet extraction at 5 μg/mL. (H, I), HSFs for standard and tablet extraction at 0.5 mg/mL. (J) Absorption spectra for zopiclone standard and tablet extraction at 5 μg/mL. (K, L), HSFs for standard and tablet extraction at 1.5 mg/mL.
